# The potential impact of insulin resistance and metabolic syndrome on migraine headache characteristics

**DOI:** 10.1186/s12883-022-02966-x

**Published:** 2022-11-12

**Authors:** Mona Ali, Mona Hussein, Rehab Magdy, Ahmed Khamis, Salsabil Abo Al-Azayem, Asmaa M Othman, Aya Ahmed, Wesam Osama

**Affiliations:** 1 Department of Neurology, Beni-Suef University, Beni-Suef, Egypt; 2 Department of Neurology, Cairo University, Cairo, Egypt; 3 Beni-Suef General Hospital, Beni-Suef, Egypt; 4 Department of Internal Medicine, Beni-suef University, Beni- Suef, Egypt; 5 Department of Clinical and Chemical Pathology, Beni-suef University, Beni-Suef, Egypt

**Keywords:** Migraine, Insulin resistance, Metabolic syndrome, MIGSEV, Headache impact Test-6

## Abstract

**Background & objectives:**

Studying comorbidities with migraine aids in a better understanding of its pathophysiology and potential therapeutic targets. This case-control study aimed to study the impact of insulin resistance and metabolic syndrome on the characteristics of migraine headache attacks.

**Methods:**

A case-control study was conducted on 30 migraine patients and 30 healthy controls. The following data were assessed in migraine patients: type of migraine, duration of attacks, Migraine Severity Scale (MIGSEV), and Headache Impact Test-6 (HIT-6). Both groups were assessed for waist circumference and underwent the following tests: fasting blood glucose, fasting insulin, high-density lipoprotein cholesterol level, and triglycerides, and homeostasis model assessment–insulin resistance (HOMA-IR) was applied.

**Results:**

This study included age and sex-matched patients and controls. Migraine patients had significantly higher waist circumference, higher mean values of serum insulin, HOMA-IR and higher frequency of insulin resistance and metabolic syndrome than the control group (*P*-value = 0.005, 0.049, 0.01, 0.012, 0.024, respectively). Migraine patients with insulin resistance had significantly higher intensity and tolerability scores, MIGSEV total score, and HIT-6 total score compared to those without (*P*-value = 0.005, 0.005, 0.002, 0.018, respectively). There was a significantly positive correlation between the MIGSEV and HIT-6 scores and fasting insulin levels, and HOMA-IR value (*P*-value = 0.006, ≤ 0.001, 0.017, ≤ 0.001, respectively).

**Conclusion:**

Insulin resistance and metabolic syndrome are more common in migraine patients than in healthy controls. The severity and impact of migraine attacks are higher in patients with insulin resistance than in those without.

## Introduction

In 2019, by the Global Burden of Disease (GBD) study, migraine was declared the second cause of disability and the first among young females [1]. Studying the comorbid conditions commonly encountered with migraine and their effect on migraine severity aids in a better understanding of the pathophysiology of migraine and the potential therapeutic targets [2].

Insulin resistance (IR) is defined as an impaired response of target cells to insulin. Different mechanisms could be related to Insulin sensitivity, including insulin receptors downregulation, the inability of insulin receptors to bind to insulin, or abnormal insulin signalling cascade activation [3]. Insulin resistance causes impairment of glucose metabolism leading to a compensatory increase in insulin production and hyperinsulinemia.

By time, insulin resistance can lead to metabolic syndrome [4]. There is increasing evidence that insulin resistance underlies the pathogenesis of abdominal obesity, diabetes, and hypertension, which are components of metabolic syndrome.

Some studies have shown that insulin resistance is related to migraine [5, 6]. Yet such an association was not ascertained by S Sacco et al. [7] and RK Özcan and SG Özmen [8]. The components of metabolic syndrome, such as hypertension, diabetes, and obesity, were also common in patients with migraine [5, 8, 9]. Epidemiological data estimated a 1-year prevalence of migraine in metabolic syndrome to be 11.9% in men and 22.5% in women [10]. However, the exact relationship is still unclear, and their relation to migraine characteristics is still not well established [11].

Accordingly, this case-control study aimed to study the impact of insulin resistance and metabolic syndrome on the characteristics of migraine headache attacks.

## Methods

### Study population

The present case-control study was conducted on 30 newly diagnosed migraine patients fulfilling ICHD-3 criteria for migraine [12] and aged 18–50 years. The patients were consecutively recruited from Beni Suef University Hospital Neurology clinic from June 1, 2021, to September 1, 2021. Another 30 age and sex-matched healthy relatives of those patients were enrolled as controls during the same period to ensure the nearly same socioeconomic level and nutritional habits between patients and the control groups.

Exclusion criteria included endocrine diseases, such as diabetes mellitus or thyroid dysfunction, and drugs with known effects on the lipid profile, insulin levels, or body weight, such as statins, fibrates, valproate, steroids, oral contraceptive pills, etc. Pregnant women were also excluded from the study.

### Procedures

Demographic data were collected from both patients and control groups. Waist circumference was also measured for patients and controls using a plastic tape measure midway between the last rib and the iliac crest at the end of normal expiration. Migraine patients were evaluated regarding the type of migraine (with or without aura), duration of untreated headache attacks, presence or absence of allodynia, and autonomic manifestations. The migraine severity was assessed based on patient self-report by the Migraine Severity Scale (MIGSEV) [13], which relies on four items: intensity of pain, disability in daily activity, tolerability, and nausea. The Headache Impact Test-6 (HIT-6) [14] was used to estimate the burden of headache, entailing six items: pain, social functioning, role functioning, vitality, cognitive functioning, and psychological distress.

Both patients and control groups underwent the following laboratory tests: fasting blood glucose (FBG), fasting insulin, high-density lipoprotein cholesterol level (HDL), and triglycerides (TGs).

Blood samples were collected by antecubital venipuncture in vacutainer tubes with Na Flouride as an anticoagulant for blood glucose and without anticoagulant for the rest of the results after fasting.

Routine chemistry investigations, including HDL-C, TG, and FBG, were analyzed on an automated Benchtop chemistry analyzer Flexor. Commercial kits were supplied by ELI Tech; ELI Tech: SEPPIM S.A.S – Zone Industrielle – 61,500 SEES FRANCE. Measuring fasting insulin level by enzyme-linked immunosorbent assay (ELISA): The kit uses a double-antibody sandwich ELISA technique.

The homeostasis model assessment–insulin resistance (HOMA-IR) was applied as follows: fasting insulin (µU/mL) × fasting glucose (mmol/L) /22.5 [15]. If the HOMA-IR value > 2.5 indicates a state of insulin resistance [16].

The National Cholesterol Education Program (NCEP) Adult Treatment Panel III (ATP III) criteria [17] necessitate at least three of the following to define metabolic syndrome: FBG ≥ 100 mg/dL, blood pressure ≥ 130/80 mmHg, a waist circumference of > 90 cm for men and > 80 cm for women, HDL cholesterol of < 40 mg/dL for men and < 50 mg/dL for women, and TGs > 150 mg/dL.

### Sample size

Before starting the study, the sample size was calculated using G*Power version 3.1.9.7 Software. The effect size was calculated based on the mean values of HOMA in migraine patients and controls in a study conducted by Fava et al. [5]. The statistical test used was the independent sample t-test. The type of power analysis was: A priori: compute required sample size- given α, power, and effect size. The input parameters were: tail(s) = two, effect size d = 1.37, α err prob = 0.05, power (1-β err prob) = 0.95, and allocation ratio N2/N1 = 1. The output parameters were: non centrality parameter δ = 3.753, critical t = 2.048, and Df = 28. A total sample size of 30 patients in both patients and control groups was required to achieve a statistical power (1–β) of 95%.

### Statistical analysis

IBM SPSS (Statistical Package of Social Science) Version 25 was used to analyze the data. Kolmogorov–Smirnov test was used to test the normality of data. Non-normally distributed quantitative variables were expressed as the median and interquartile range (IQR), while normally distributed quantitative variables were expressed as mean and standard deviation. Categorical variables were expressed as numbers and percentages. An independent sample t-test was used to compare migraine patients and the control group in quantitative normally distributed variables. In contrast, the Mann-Whitney test was used to compare the two groups in quantitative, non-normally distributed variables. Chi-squared test was used to compare migraine patients and the control group in categorical variables. Correlations between the duration and severity of migraine attacks and the laboratory markers of insulin resistance and metabolic syndrome were done using the Spearman correlation test. Multivariate regression analysis was done to detect the laboratory markers associated with increased duration and severity of migraine headache attacks. The independent variables were FBS, TGs, HDL, Insulin, and HOMA-IR. *P*-value ≤ 0.05 was considered statistically significant. All tests were two-tailed.

## Results

### Demographics, clinical and laboratory characteristics of migraine patients and controls

This case-control study was conducted on 30 migraine patients and 30 age and sex-matched controls (*P*-value = 0.734, 0.766, respectively). There was a statistically significant difference between migraine patients and controls regarding the mean value of waist circumference [92.43 ± 11.14 cm vs. 84.9 ± 8.42 cm, *P*-value = 0.005)]. Only five migraine patients (16.7%) were known to be hypertensive.

Migraine patients had significantly higher mean values of serum insulin and HOMA-IR than the control group (*P*-value = 0.049, 0.01, respectively). Whereas, there were no statistically significant differences between migraine patients and controls regarding FBS, TGs, or HDL (*P*-value = 0.719, 0.282, 0.397, respectively).

Migraine patients had a significantly higher frequency of insulin resistance (46.7%) in comparison to controls (16.7%) (*P*-value = 0.012). Also, the frequency of metabolic syndrome was significantly higher in migraine patients (43.3%) in comparison to controls (16.7%) (*P*-value = 0.024) (Table 1).

**Table 1:** Demographics, clinical and laboratory characteristics of migraine patients and controls

		Migraine patients (*n* = 30)	Controls (*n* = 30)	*P*-value
Age in years [median (IQR)]		34.5 (27–43)	36.5 (26–41)	0.734 ^1^
Sex [n (%)]	Males	8 (26.7%)	7 (23.3%)	0.766 ^2^
	Females	22 (73.3%)	23 (76.7%)	
Waist circumference in cm [mean (SD)]		92.43 (11.14)	84.9 (8.42)	0.005* ^3^
HTN [n (%)]		5 (16.7%)		
Attack duration in hours [median (IQR)]		12 (8- 13.75)		
Aura [n (%)]		7 (23.3%)		
Autonomic manifestations [n (%)]		2 (6.7%)		
Allodynia [n (%)]		9 (30%)		
MIGSEV Scale [median (IQR)]	Intensity	3 (2–3)		
	Disability	3 (2–3)		
	Tolerability	2 (1–2)		
	Nausea	2 (1–3)		
	Total score	2 (1–3)		
HIT-6 total score [median (IQR)]		59 (49–64)		
Laboratory work up [mean (SD)]	FBS	87.87 (12.19)	86.8 (10.6)	0.719 ^3^
	TGs	97.4 (49.45)	109.77 (37.97)	0.282 ^3^
	HDL	50.51 (11.25)	52.93 (10.76)	0.397 ^3^
	Insulin	7.03 (2.997)	5.37 (3.41)	0.049* ^3^
	HOMA-IR	1.72 (0.79)	1.17 (0.81)	0.01* ^3^
Insulin resistance [n (%)]		14 (46.7%)	5 (16.7%)	0.012* ^2^
Metabolic syndrome [n (%)]		13 (43.3%)	5 (16.7%)	0.024* ^2^

*FBS* Fasting blood sugar, *HDL* High-density lipoprotein, *HIT-6* Headache Impact Test-6, *HOMA-IR* Homeostatic Model Assessment for Insulin Resistance, *HTN* Hypertension, *MIGSEV* Migraine Severity Scale, *TGs* Triglycerides

^1^Mann-Whitney test, ^2^Chi-squared test, ^3^Independent sample t-test

*P-value ≤ 0.05 is considered significant

### Characteristics of migraine headache attacks in the included migraine patients

The median value for the duration of untreated migraine headache attacks was 12 h with IQR (8- 13.75). Only 23.3% of migraine patients had an aura, 6.7% had autonomic manifestations, and 30% had allodynia. The median value for MIGSEV total score was 2 with IQR (1–3), while the median value for the HIT-6 total score was 59 with IQR (49–64) (Table 2).

**Table 2:** Characteristics of migraine in patients with and without insulin resistance

		Migraine patients with insulin resistance (n = 14)	Migraine patients without insulin resistance (n = 16)	*P*-value
Attack duration in hours [median (IQR)]		12 (11–14)	9 (6.5–15)	0.22 ^1^
Aura [n (%)]		3 (21.4%)	4 (25%)	0.818 ^2^
Autonomic manifestations [n (%)]		1 (7.1%)	1 (6.3%)	0.922 ^2^
Allodynia [n (%)]		4 (28.6%)	5 (31.3%)	0.873 ^2^
MIGSEV Scale [median (IQR)]	Intensity	3 (3–4)	2 (2–3)	0.005* ^1^
	Disability	3 (3–3)	2.5 (2–3)	0.12 ^1^
	Tolerability	2 (2–3)	2 (1–2)	0.005* ^1^
	Nausea	2.5 (2–4)	2 (1–3)	0.188 ^1^
	Total score	3 (2–3)	1.5 (1- 2.75)	0.002* ^1^
HIT-6 total score [median (IQR)]		63 (58.25–64.25)	55.5 (42- 61.25)	0.018* ^1^

*HIT* Headache Impact Test-6, *MIGSEV* Migraine Severity Scale

^1^Mann-Whitney test, ^2^Chi-squared test

*P-value ≤ 0.05 is considered significant

### The impact of insulin resistance and metabolic syndrome on the characteristics of migraine headache attacks

Migraine patients with insulin resistance had significantly higher intensity and tolerability scores, MIGSEV total score, and HIT-6 total score compared to those without insulin resistance (*P*-value = 0.005, 0.005, 0.002, 0.018, respectively). Whereas, there were no statistically significant differences between migraine patients with and without insulin resistance regarding the duration of untreated migraine headache attacks, aura, autonomic manifestations, allodynia, disability score, or nausea (Table 2).

There were no statistically significant differences between migraine patients with and without metabolic syndrome regarding the duration of untreated migraine headache attacks, aura, autonomic manifestations, allodynia, intensity, disability or tolerability scores, nausea, MIGSEV total score, or HIT-6 total score (Table 3).

**Table 3:** Characteristics of migraine in patients with and without metabolic syndrome

		Migraine patients with metabolic syndrome (*n* = 13)	Migraine patients without metabolic syndrome (*n* = 17)	*P*-value
Attack duration in hours [median (IQR)]		12 (8- 17.5)	12 (7.5–12)	0.259 ^1^
Aura [n (%)]		2 (15.4%)	5 (29.4%)	0.368 ^2^
Autonomic manifestations [n (%)]		1 (7.7%)	1 (5.9%)	0.844 ^2^
Allodynia [n (%)]		5 (38.5%)	4 (23.5%)	0.367 ^2^
MIGSEV Scale [median (IQR)]	Intensity	3 (2- 3.5)	3 (2–3)	0.807 ^1^
	Disability	3 (2–3)	3 (2–3)	0.657 ^1^
	Tolerability	2 (1.5–2.5)	2 (1- 2.5)	0.82 ^1^
	Nausea	2 (1.5- 4)	2 (1–3)	0.529 ^1^
	Total score	3 (1.5-3)	2 (1–3)	0.26 ^1^
HIT-6 total score [median (IQR)]		63 (48–64)	59 (47- 62.5)	0.628 ^1^

*HIT* Headache Impact Test-6, *MIGSEV* Migraine Severity Scale

^1^Mann-Whitney test, ^2^Chi-squared test

*P*-value > 0.05 is considered non-significant

There was a statistically significant negative correlation between the duration of untreated migraine headache attacks and HDL (*P*-value = 0.002). There were statistically significant positive correlations between MIGSEV total score and FBS, insulin, and HOMA-IR serum levels (*P*-value = 0.016, 0.006, ≤ 0.001 respectively), and also between HIT-6 total score, and both insulin and HOMA-IR serum levels (*P*-value = 0.017, ≤ 0.001 respectively) (Table 4).

**Table 4:** Correlations between duration and severity of migraine attacks and the laboratory markers of insulin resistance and metabolic syndrome

		Attack duration	MEGSEV	HIT	FBS	TGs	HDL	Insulin	HOMA-IR
Attack duration	(r) Coef.	1	0.333	0.366	0.148	0.077	-0.534	0.134	0.299
	*P*-value	.	0.072	0.047*	0.434	0.686	0.002*	0.482	0.108
MEGSEV	(r) Coef.	0.333	1	0.705	0.437	0.302	-0.164	0.486	0.721
	*P*-value	0.072	.	< 0.001*	0.016*	0.104	0.387	0.006*	< 0.001*
HIT	(r) Coef.	0.366	0.705	1	0.325	0.295	-0.026	0.433	0.704
	*P*-value	0.047*	< 0.001*	.	0.08	0.114	0.891	0.017*	< 0.001*
FBS	(r) Coef.	0.148	0.437	0.325	1	0.521	-0.282	0.358	0.390
	*P*-value	0.434	0.016*	0.08	.	0.003*	0.131	0.052	0.033*
TGs	(r) Coef.	0.077	0.302	0.295	0.521	1	-0.213	0.268	0.317
	*P*-value	0.686	0.104	0.114	0.003*	.	0.258	0.152	0.088
HDL	(r) Coef.	-0.534	-0.164	-0.026	-0.282	-0.213	1	0.111	0.071
	*P*-value	0.002*	0.387	0.891	0.131	0.258	.	0.559	0.708
Insulin	(r) Coef.	0.134	0.486	0.433	0.358	0.268	0.111	1	0.813
	*P*-value	0.482	0.006*	0.017*	0.052	0.152	0.559	.	< 0.001*
HOMA-IR	(r) Coef.	0.299	0.721	0.704	0.390	0.317	0.071	0.813	1
	*P*-value	0.108	< 0.001*	< 0.001*	0.033*	0.088	0.708	< 0.001*	.

*FBS* Fasting blood sugar, *HDL* High-density lipoprotein, *HIT* Headache Impact Test-6, *HOMA-IR* Homeostatic Model Assessment for Insulin Resistance, *MIGSEV* Migraine Severity Scale, *TGs* Triglycerides

(r): Spearman coefficient, *P-value ≤ 0.05 is considered significant

Multivariate regression analysis was done to detect the laboratory markers associated with increased duration and severity of migraine headache attacks. The independent variables were FBS, TGs, HDL, Insulin, and HOMA-IR.

HDL was found to be associated with increased duration of migraine headache attacks (*P*-value = 0.039), whereas HOMA-IR was associated with increased MIGSEV and HIT-6 total scores (*P*-value ≤ 0.001, 0.002, respectively) (Table 5).

**Table 5:** Multivariate regression analysis to detect the laboratory markers associated with increased duration and severity of migraine headache attacks

		Unstandardized Coefficients		Standardized Coefficients	t	*P*-value	95.0% Confidence Interval for B		Collinearity Statistics	
		B	SE	Beta			Lower Bound	Upper Bound	Tolerance	VIF
Attack duration^a^	Constant	31.395	12.232		2.567	0.017	6.150	56.640		
	FBS	-0.073	0.112	-0.133	-0.651	0.521	-0.305	0.159	0.747	1.339
	TGs	-0.025	0.029	-0.187	-0.863	0.397	-0.086	0.035	0.665	1.505
	HDL	-0.267	0.122	-0.448	-2.182	0.039*	-0.519	-0.015	0.740	1.351
	Insulin	-0.833	0.788	-0.372	-1.057	0.301	-2.460	0.794	0.251	3.981
	HOMA-IR	5.356	2.904	0.627	1.844	0.078	-0.638	11.350	0.269	3.715
MEGSEV^b^	Constant	0.297	1.087		0.273	0.787	-1.946	2.540		
	FBS	0.016	0.010	0.225	1.567	0.130	-0.005	0.036	0.747	1.339
	TGs	-0.001	0.003	-0.081	-0.529	0.602	-0.007	0.004	0.665	1.505
	HDL	-0.009	0.011	-0.119	-0.828	0.416	-0.031	0.013	0.740	1.351
	Insulin	-0.106	0.070	-0.374	-1.511	0.144	-0.250	0.039	0.251	3.981
	HOMA-IR	1.083	0.258	1.005	4.197	<0.001*	0.550	1.615	0.269	3.715
HIT^c^	Constant	32.816	15.281		2.148	0.042	1.278	64.354		
	FBS	0.146	0.140	0.178	1.037	0.310	-0.144	0.435	0.747	1.339
	TGs	0.013	0.037	0.066	0.362	0.720	-0.062	0.089	0.665	1.505
	HDL	0.017	0.153	0.019	0.110	0.913	-0.299	0.332	0.740	1.351
	Insulin	-1.813	0.985	-0.544	-1.841	0.078	-3.846	0.219	0.251	3.981
	HOMA-IR	12.623	3.628	0.993	3.479	0.002*	5.134	20.111	0.269	3.715

*FBS* Fasting blood sugar*, HDL *High-density lipoprotein, *HIT* Headache Impact Test-6, *HOMA-IR* Homeostatic Model Assessment for Insulin Resistance, *MIGSEV *Migraine Severity Scale, *TGs *Triglycerides*. *

^a^Adjusted R square=0.097, ^b^Adjusted R square= 0.553, ^c^Adjusted R square= 0.364

*P-value ≤ 0.05 is considered significant

## Discussion

The impairment of glucose metabolism in migraine is an imperative topic of interest. Recently, EC Gross et al. [18] described migraine as an adaptive response in genetically predisposed individuals with unbalanced cerebral energy metabolism.

The current study confirmed the presence of a significant relationship between migraine and insulin resistance; the migraine group had a significantly higher frequency of insulin resistance than normal controls. Similarly, HOMA-1R was employed to measure insulin resistance in the migraine research field by SK Bhoi et al. [9], A Fava et al. [5], S Sacco et al. [7], and RK Özcan and SG Özmen [8]. Nevertheless, only two former studies found an association between migraine and insulin resistance. On the other hand, other studies have obtained similar results using different assessment methods of insulin resistance other than the HOMA-1R technique [6, 19].

Concerning these previously published reports, the impact of insulin resistance on the severity and duration of headache attacks was only studied by SK Bhoi et al. [9]. Contrary to our findings, SK Bhoi et al. [9] found that the duration of attacks was significantly longer in migraine patients with insulin resistance than in those without insulin resistance, but no significant difference regards headache severity. At the same time, the current study showed a significantly positive correlation between the MIGSEV and HIT-6 scores and fasting insulin levels, and HOMA-IR value.

At the brain level, insulin resistance is proposed to lead to impaired neurotransmitter release, altered neuronal and glial cell receptor regulation, or homeostatic and inflammatory responses to insulin. All these mechanisms are proposed to be hypothetical models for the relation of insulin resistance and metabolic syndrome to migraine [11, 20].

In light of these observations, B Guldiken et al. [10] endorsed careful assessment for impaired glucose metabolism in patients with migraine. Various methods have emerged to correct insulin resistance or hyperinsulinism, such as insulin-sensitizing drugs, physical activity, and dietary interventions [21, 22]. Ultimately, these therapeutic modalities are worthy of being evaluated from this perspective to assess their effects on migraine.

In agreement with previous studies [9, 10], this study showed a statistically significant higher frequency of metabolic syndrome in the migraine group than in normal controls. Yet, no significant effect of metabolic syndrome was detected on the severity or duration of migraine attacks. In contrast, SK Bhoi et al. [9] found that the duration of attacks was significantly longer in migraine patients with metabolic syndrome than in those without, but no significant difference regards headache severity.

Regarding different components of metabolic syndrome, this study showed a significantly higher waist circumference in migraine patients than in normal controls. The relation between migraine and abdominal obesity was also stated in previous reports [23, 24]. Adipose tissue is largely accepted as an endocrine gland from which many pro-inflammatory cytokines can be produced, including IL-6 and TNF-α [24]. Their levels increase just before initiating a migraine attack [25].

The association between dyslipidemia and migraine is controversial. Some studies have shown a higher frequency of dyslipidemia in migraine than in healthy controls [19, 26], while others did not find such a relationship [8, 9]. Although this study did not find a statistically significant difference between migraine and normal controls regarding TGs and HDL levels, a negative correlation was observed between decreased HDL levels and increased headache duration. Z Bic et al. [27] proposed that elevated blood lipids might lead to increased platelet aggregation, decreased serotonin levels, and elevated prostaglandin levels, causing vasodilatation that heralds a migraine attack.

This study had some limitations. First, a small sample size hindered further comparative analysis between migraine subgroups (migraine with aura vs. migraine without aura). Second, its cross-sectional design in which the exact sequence of the medical events could not be recognized.

## Conclusion

Insulin resistance and metabolic syndrome are more common in migraine patients than in healthy controls. The severity and impact of migraine headache attacks are higher in patients with insulin resistance than in those without insulin resistance. At the same time, they are not different in patients with and without metabolic syndrome. The higher the HOMA-IR value level, the more severe the migraine headache attacks.

## Data Availability

Authors report that the datasets used and/or analyzed during the current study are available from the corresponding author upon reasonable request.
